# Supplementary material for: Prevalence and risk factors of Salmonella in commercial poultry farms in Nigeria
Source: PLoS One. 2020 Sep 23;15(9):e0238190. doi: 10.1371/journal.pone.0238190 (PMC7510976; doi:10.1371/journal.pone.0238190)
Supplement: S1 Table — (DOCX) [file pone.0238190.s001.docx]

**S1 Table: Quality assurance of 89 genomes assemblies for inclusion into the study**

| **Reason for exclusion** | **Count** | **Percentage** |
| --- | --- | --- |
| Contamination | 5 | 6 |
| Assignment to different organism | 4 | 4 |
| Genome size | 3 | 3 |
| GC Content | 3 | 3 |
| **Cumulative fail** | 15 | 17 |
