# Supplementary material for: Prevalence and risk factors of Salmonella in commercial poultry farms in Nigeria
Source: PLoS One. 2020 Sep 23;15(9):e0238190. doi: 10.1371/journal.pone.0238190 (PMC7510976; doi:10.1371/journal.pone.0238190)
Supplement: S2 Table — (DOCX) [file pone.0238190.s002.docx]

**S2 Table: Genomic characteristics and serotype predictions of *Salmonella* strains isolated from poultry in Nigeria**

| **ID** | **Genome size (bp)** | **N50 (bp)** | **GC Content (%)** | **SeqSero 2 prediction** | | **SISTR prediction** | |  | **Serotype based on**  **PCR** |
| --- | --- | --- | --- | --- | --- | --- | --- | --- | --- |
|  |  |  |  | **Predicted**  **serotype** | **Antigenic phenotype** | **Predicted**  **serotype** | **Antigenic phenotype** | **ST** |  |
| A5 | 4614052 | 175414 | 52.2 | Schwarzengrund | 4:d:1,7 | Schwarzengrund | B:d:1,7 | 96 | - |
| A12 | 4804758 | 228730 | 52.2 | Kentucky | 8:i:z6 | Kentucky | C2-C3:i:z6 | 198 | Enteritidis |
| A15 | 4782801 | 36492 | 52.3 | Kentucky | 8:i:z6 | Kentucky | C2-C3:i:z6 | 198 | - |
| A16 | 4833880 | 70317 | 52.2 | Kentucky | 8:i:z6 | Kentucky | C2-C3:i:z6 | 198 | - |
| A18 | 4950166 | 52261 | 52.1 | Not in WKL | -:d:1,5 | Isangi | C1:d:1,5 | 216 | - |
| A24 | 4684723 | 109603 | 52.2 | Not in WKL | :r:1,2 | Colindale | C1:r:1,7 | 584 | - |
| A25 | 4615016 | 163184 | 52.3 | Chester | 4:e,h:e,n,x | Chester | B:e,h:e,n,x | 411 | Enteritidis |
| A27 | 4764221 | 49507 | 52.3 | Not in WKL | 9,46:i:1,2 | *Aberdeen | :i:1,2 | ¤3320 | Enteritidis |
| A28 | 4724411 | 36847 | 52.3 | Not in WKL | :e,h:1,5 | Muenster | :e,h:1,5 | 321 | - |
| A29 | 4835154 | 177057 | 52.2 | Kentucky | 8:i:z6 | Kentucky | C2-C3:i:z6 | 198 | - |
| A31 | 4718012 | 436787 | 52.2 | Schwarzengrund | 4:d:1,7 | Schwarzengrund | B:d:1,7 | 96 | - |
| A32 | 4670095 | 183138 | 52.2 | Give | 3,10:l,v:1,7 | Give | E1:l,v:1,7 | 524 | - |
| A34 | 4686582 | 367558 | 52.3 | Isangi | 30:d:1,5 | Isangi | C1:d:1,5 | 216 | Enteritidis |
| A35 | 4709441 | 159719 | 52.3 | Isangi | 30:d:1,5 | Isangi | C1:d:1,5 | 216 | - |
| A37 | 4581012 | 176404 | 52.2 | Chester | 4:e,h:e,n,x | Chester | B:e,h:e,n,x | 411 | Enteritidis |
| A39 | 4940990 | 146967 | 52.2 | Kentucky | 8:i:z6 | Kentucky | C2-C3:i:z6 | 198 | - |
| A40 | 4826780 | 68784 | 52.2 | Kentucky | 8:i:z6 | Kentucky | C2-C3:i:z6 | 198 | Enteritidis |
| A44 | 4677596 | 284222 | 52.2 | Virchow | 7:r:1,2 | Virchow | C1:r:1,2 | ¤6166 | - |
| A48 | 4602682 | 163734 | 52.2 | Ituri | 4:z10:1,5 | Ituri | B:z10:1,5 | ¤4498 | - |
| A51 | 4670152 | 226163 | 52.2 | Schwarzengrund | 4:d:1,7 | Schwarzengrund | B:d:1,7 | 96 | - |
| A52 | 4746380 | 254254 | 52.2 | Waycross | 41:z4:z23:- | Waycross | S:z4:z23:- | ¤7745 | - |
| A53 | 4673207 | 228350 | 52.2 | Ituri | 4:z10:1,5 | Ituri | B:z10:1,5 | ¤4498 | - |
| A54 | 4945658 | 38852 | 52.1 | Isangi | 30:d:1,5 | Isangi | C1:d:1,5 | 216 | - |
| A55 | 4772497 | 118677 | 52.1 | Muenster | -:e,h:1,5 | Muenster | E1:e,h:1,5 | 321 | - |
| A56 | 4827687 | 95162 | 52.2 | Kentucky | 8:i:z6 | Kentucky | C2-C3:i:z6 | 198 | - |
| A59 | 4762869 | 89970 | 52.2 | Muenster | 3,10:e,h:1,5 | Muenster | E1:e,h:1,5 |  | - |
| A61 | 4799827 | 46066 | 52.2 | Kentucky | 8:i:z6 | Kentucky | C2-C3:i:z6 | 198 | - |
| A69 | 4833962 | 95739 | 52.2 | Kentucky | 8:i:z6 | Kentucky | C2-C3:i:z6 | 198 | - |
| A71 | 4609168 | 85855 | 52.2 | Schwarzengrund | 4:d:1,7 | Schwarzengrund | B:d:1,7 | 96 | - |
| A72 | 4688214 | 29925 | 52.4 | Not in WKL | :e,h:1,5 | Muenster | E1:e,h:1,5 | 321 | - |
| B4 | 4773471 | 122648 | 52.2 | Kentucky | 8:i:z6 | Kentucky | C2-C3:i:z6 | 198 | Enteritidis |
| B5 | 4857427 | 125711 | 52.2 | Kentucky | 8:i:z6 | Kentucky | C2-C3:i:z6 | 198 | - |
| B12 | 4650554 | 85581 | 52.2 | Takoradi | 8:i:1,5 | Takoradi | C2-C3:i:1,5 | 531 | - |
| B13 | 4516132 | 89826 | 52.4 | Poona | 13:z:1,6 | Poona | G:z:1,6 | 308 | - |
| B14 | 4822613 | 32863 | 52.3 | Not in WKL | :i:z6 | Kentucky | C2-C3:i:z6 | 198 | Enteritidis |
| B16 | 4879536 | 163925 | 52.2 | Kentucky | 8:i:z6 | Kentucky | C2-C3:i:z6 | 198 | - |
| B17 | 4932397 | 168366 | 52.2 | Kentucky | 8:i:z6 | Kentucky | C2-C3:i:z6 | 198 | - |
| B20 | 4741351 | 26266 | 52.4 | Takoradi | :i:1,5 | Takoradi | C2-C3:i:1,5 | 531 | - |
| B21 | 4771969 | 167116 | 52.2 | Takoradi | :i:1,5 | Takoradi | C2-C3:i:1,5 | 531 | Enteritidis |
| B26 | 4791495 | 36853 | 52.3 | Kentucky | 8:i:z6 | Kentucky | C2-C3:i:z6 | 198 | - |
| B27 | 4807365 | 314557 | 52.0 | Corvallis | 8:z4,z23:- | Corvallis | C2-C3:z4,z23:- | ¤7744 | - |
| B28 | 4782264 | 142784 | 52.2 | Takoradi | 8:i:1,5 | Takoradi | C2-C3:i:1,5 | 531 | - |
| B31 | 4668656 | 503991 | 52.2 | Takoradi | 8:i:1,5 | Takoradi | C2-C3:i:1,5 | 531 | - |
| B35 | 4833763 | 79845 | 52.2 | Not in WKL | -:i:z6 | Kentucky | C2-C3:i:z6 | 198 | - |
| B36 | 4776436 | 82664 | 52.2 | Takoradi | 8:i:1,5 | Takoradi | C2-C3:i:1,5 | 531 | - |
| C1 | 4931250 | 369152 | 52.1 | Kentucky | 8:i:z6 | Kentucky | C2-C3:i:z6 | 198 | - |
| C3 | 4784805 | 193841 | 52.2 | Chomedey | 8:z10:e,n,z15 | Chomedey | C2-C3:z10:e,n,z15 | ¤3961 | - |
| C5 | 4798387 | 120098 | 52.1 | Alachua | 35:z4,z23:- | Alachua | O:z4,z23:- | ¤7743 | - |
| C6 | 4771463 | 101600 | 52.2 | Hato | 4:g,m,s:- | Essen\|Hato | B:g,m,s:- | ¤7747 | - |
| C10 | 4869231 | 199356 | 52.2 | Menston | 7:g,s,t:- | Menston | C1:g,[s],t:- | ¤7742 | - |
| C11 | 4683225 | 97334 | 52.3 | Not in WKL | :d:1,5 | Isangi | C1:d:1,5 | 216 | - |
| C12 | 4782452 | 153493 | 52.1 | Bradford | 4:r:1,5 | Bradford | B:r:1,5 | ¤7746 | - |
| C13 | 4837420 | 228679 | 52.2 | Kentucky | 8:i:z6 | Kentucky | C2-C3:i:z6 | 198 | - |
| C14 | 4638064 | 21684 | 52.5 | Not in WKL | :g,m,s:- | ¶Abadina | :g,m,s:- | ¤3899 | - |
| C15 | 4976217 | 58440 | 52.0 | Isangi | 30:d:1,5 | Isangi | C1:d:1,5 | 216 | - |
| C16 | 4752039 | 29180 | 52.3 | Corvallis | 8:z4,z23:- | Corvallis | C2-C3:z4,z23:- | ¤7744 | Enteritidis |
| C17 | 4820683 | 535385 | 52.2 | Kentucky | 8:i:z6 | Kentucky | C2-C3:i:z6 | 198 | - |
| C24 | 5011327 | 12007 | 52.3 | Isangi | 30:d:1,5 | Isangi | C2-C3:i:z6 | 216 | - |
| C26 | 4844648 | 31407 | 52.2 | Not in WKL | :d:I,w | Birmingham§ |  | ¤7749 | Enteritidis |
| C28 | 4569280 | 32414 | 52.5 | Not in WKL | -:z13,z28:I,z13,z28 | No prediction | -:z13,z28:I,z13,z28 | - | - |
| C29 | 4806860 | 101789 | 52.3 | Kentucky | 8:i:z6 | Kentucky | C2-C3:i:z6 | 198 | - |
| C30 | 4812453 | 461050 | 52.2 | Kentucky | 8:i:z6 | Kentucky | C2-C3:i:z6 | 198 | - |
| C31 | 4776546 | 97692 | 52.2 | Kentucky | 8:i:z6 | Kentucky | C2-C3:i:z6 | 198 | - |
| C33 | 4762453 | 49072 | 52.2 | Not in WKL | :e,h:1,2 | Larochelle | C1:e,h:1,2 | 22 | - |
| C34 | 4790944 | 118348 | 52.1 | Not in WKL | :e,h:1,2 | Larochelle | C1:e,h:1,2 | 22 | - |
| C36 | 5000154 | 31754 | 52.1 | Kentucky | 8:i:z6 | Kentucky | C2-C3:i:z6 | 198 | - |
| C37 | 4812791 | 390253 | 52.1 | Larochelle | -:e,h:1,2 | Larochelle | C1:e,h:1,2 | 22 | Enteritidis |
| C39 | 4672849 | 32932 | 52.4 | Not in WKL | :g,m,s:- | ¶Abadina | :g,m,s:- | ¤3899 | - |
| C44 | 4835990 | 484201 | 52.2 | Kentucky | 8:i:z6 | Kentucky | C2-C3:i:z6 | 198 | - |
| C45 | 4456070 | 17293 | 52.6 | Not in WKL | :d:e,n,z15 | Telelkebir | -:d:e,n,z15 | 2222 | - |
| C46 | 4974476 | 322485 | 52.0 | Isangi | 30:d:1,5 | Isangi | C1:d:1,5 | 216 | - |
| C50 | 4823401 | 200805 | 52.2 | Larochelle | -:e,h:1,2 | Larochelle | C1:e,h:1,2 | 22 | - |
| C52 | 4508753 | 142350 | 52.3 | Telelkebir | -:d:e,n,z15 | Telelkebir | G:d:e,n,z15 | 2222 | Enteritidis |
| C53 | 4458475 | 32626 | 52.5 | Not in WKL | :d:e,n,z15 | Telelkebir | -:d:e,n,z15 | 2222 | - |

¤ STs assigned by Enterobase

*Aberdeen|Augustenborg|Cuckmere|Diourbel|Doorn|Landau|Lindenburg|Mbao|Mgulani|Stratford|Typhimurium

¶Abadina|Adeoyo|Agbeni|Amsterdam|Bijlmer|Blegdam|Bron|Caracas|Chincol|Croft|Dublin|Ealing|Emek|Enteritidis|Essen|Gamaba|Giessen|Godesberg|Gueuletapee|Hato|Hillingdon|Kiel|Korovi|Kouka|Luke|Montevideo|Moscow|Naestved|Nikolaifleet|Nitra|Othmarschen|Plumaugat|Pontypridd|Rostock|Suberu|Sylvania|Tornow|Warragul

§|Caen|Cullingworth|Dembe|Livingstone|Mons|Niamey|Putten|Tilburg

Simpson diversity index = 0.87

WKL = White Kauffman Le-Minor
