# Supplementary material for: Prevalence and risk factors of Salmonella in commercial poultry farms in Nigeria
Source: PLoS One. 2020 Sep 23;15(9):e0238190. doi: 10.1371/journal.pone.0238190 (PMC7510976; doi:10.1371/journal.pone.0238190)
Supplement: S3 File — (DOCX) [file pone.0238190.s005.docx]

**Questionnaire Survey on the Risk Factors of Salmonella infection in commercial poultry farms in Northwest, Nigeria**

**1.0 General Farm Data**

| 1.1 | Date of Survey (dd/mm/yy) |  |
| --- | --- | --- |
| 1.2 | Name of Investigator |  |
| 1.3 | Name of farm/farm ID |  |
| 1.4 | Local Government and State |  |
| 1.5 | GPS coordinate | Latitude: Longitude: |
| 1.6 | Farm Category | **󠆹**Backyard **󠆹󠆹**Semi commercial **󠆹󠆹**Small scale **󠆹󠆹**Medium scale **󠆹󠆹**Large scale |

**2.0 Farmer/Farm Manager Demography**

| 2.1 | Age | **󠆹** 20-30 **󠆹󠆹** 31-40 **󠆹󠆹**41-50 **󠆹󠆹**above 50 |
| --- | --- | --- |
| 2.2 | Gender | **󠆹** Male **󠆹󠆹** Female |
| 2.3 | Marital Status | **󠆹** Married **󠆹󠆹** Single |
| 2.4 | Education | **󠆹**Informal/adult education **󠆹󠆹** Primary education **󠆹󠆹** Secondary education **󠆹󠆹**Tertiary education |
| 2.5 | Profession | **󠆹**Poultry farmer **󠆹󠆹**Civil servant  **󠆹**Private sector **󠆹󠆹**others, specify………..... |
| 2.6 | How long have you been a poultry farmer | **󠆹**  < 1 year **󠆹󠆹**1- 5 years **󠆹󠆹**5-10 years **󠆹󠆹** > 10 years |
| 2.7 | Contact with animal health or extension worker | **󠆹** Yes **󠆹󠆹**No |
| 2.8 | Mobile phone number |  |

**3.0 Farm size and management**

| **3.1** | Flock size |  |
| --- | --- | --- |
| **3.2** | Breed of Flock |  |
| **3.3** | Age of flock, category (weeks) |  |
| **3.4** | Source of Day old chick (DOC) | **󠆹**Agrited **󠆹󠆹**Amo **󠆹󠆹** ChiFarms **󠆹󠆹** Farm Support **󠆹󠆹**Olam **󠆹󠆹**Royal Farm **󠆹󠆹**Yamfi farm **󠆹**Other specify |
| **3.5** | System of production | **󠆹**Deep Litre **󠆹󠆹**Battery Cage |
| **3.6** | Source of water | **󠆹**Well water **󠆹󠆹** Borehole **󠆹󠆹**Tap water |

**4.0 Knowledge on Salmonellosis**

| 4.1 | Are you aware of Fowl Typhoid/Pullorum in poultry | **󠆹**Yes **󠆹󠆹**No |
| --- | --- | --- |
| 4.2 | Do you know it is caused by *Salmonella* | **󠆹**Yes **󠆹󠆹**No |
| 4.3 | Have you ever had Salmonella infection in your farm | **󠆹**Yes **󠆹󠆹**No |
| 4.4 | If yes, what categories of birds are most affected | **󠆹**Layer Chicks **󠆹󠆹**Pullets **󠆹**Layers **󠆹󠆹**Broiler chicks **󠆹**Broilers |
| 4.5 | If yes in (4.3) what are the common symptoms you observed | **󠆹**Diarrhoea **󠆹󠆹** Pasty vent **󠆹**Decrease egg production **󠆹**Mortality |
| 4.6 | How many episode of Salmonella in the last three years | **󠆹**Once **󠆹󠆹**Twice **󠆹󠆹** More, Specify ----------- |
| 4.7 | In percentage estimate, how many of your birds were affected | **󠆹**Less than 10 % **󠆹󠆹**11-30 % **󠆹**  **󠆹**31- 60 % **󠆹󠆹**above 60 % |
| 4.8 | In percentage estimates, how many of the affected died of the infection | **󠆹**Less than 10 % **󠆹󠆹**11-30 % **󠆹**  **󠆹**31- 60 % **󠆹󠆹**above 60 % |

**5.0 Disease Management**

| 5.1 | How did you diagnose Salmonella infection | **󠆹**Based on signs **󠆹󠆹**Laboratory confirmation **󠆹**Base on experience |
| --- | --- | --- |
| 5.2 | How do you manage outbreak | **󠆹**Isolate and treat **󠆹󠆹**Treat within the flock **󠆹**Treat all the flock **󠆹󠆹**Sale **󠆹󠆹**others, specify________ |
| 5.3 | During outbreak, is there report of neighbouring farm experiencing outbreaks? | **󠆹**Yes **󠆹󠆹**No |
| 5.4 | Do you consider management of Salmonella infection problematic | **󠆹**Yes **󠆹󠆹**No |
| 5.5 | Do you have keep records of disease outbreaks | **󠆹**Yes **󠆹󠆹**No |
| 5.6 | Do you submit monthly disease report to the State Ministry | **󠆹**Yes **󠆹󠆹**No |

**6.0 Farm Sanitation and Biosecurity**

| **6.1** | Farm fenced | **󠆹**Yes **󠆹󠆹** No |
| --- | --- | --- |
| **6.2** | Floor type | **󠆹**Concrete **󠆹󠆹**others |
| **6.3** | Poultry waste management | **󠆹**On farm **󠆹󠆹**off-farm |
| **6.4** | Poultry by-product disposal | **󠆹**On-farm **󠆹󠆹**Off-farm |
| **6.5** | Rodent control | **󠆹**Yes **󠆹󠆹**No |
| **6.6** | Present of other livestock on farm | **󠆹**Yes **󠆹󠆹** No |
| **6.7** | Presence of other poultry farm within 1 km radius | **󠆹**Yes **󠆹󠆹** No |
| **6.8** | Provision of foot dip | **󠆹**Yes **󠆹󠆹** No |
| **6.9** | Provision of lavatory | **󠆹**Yes **󠆹󠆹**No |
| **6.10** | How often do you clean poultry house | **󠆹**Never **󠆹󠆹**rarely **󠆹󠆹**Sometimes **󠆹**Most time |
| **6.11** | How do you clean poultry pen after depopulation | **󠆹**With Disinfectant **󠆹󠆹**Water **󠆹**Detergents **󠆹󠆹**Not necessary |
| **6.12** | Methods of disposal of dead carcass | **󠆹**Incineration **󠆹󠆹**Bury **󠆹󠆹**Refuse dumping **󠆹󠆹**Consume |

**7.0 Awareness on the zoonotic potentials**

| **7.1** | Do you know that humans can get infected with *Salmonella* | **󠆹**Yes **󠆹󠆹**No |
| --- | --- | --- |
| **7.2** | If Yes, how does human get infected | **󠆹**Through consumption of infected chickens **󠆹󠆹**Contact with sick chickens |
| **7.3** | If Yes, has there being report of staff having diarrhoea during outbreaks | **󠆹**Yes **󠆹󠆹** No |

**PRO FORMA FOR MOLECULAR EPIDEMIOLOGY AND ANTIMICROBIAL RESISTANCE OF *SALMONELLA ENTERICA* IN POULTRY AND HUMANS, NORTHWESTERN NIGERIA**

**(PARTICIPANT INFORMATION SHEET AND CONSENT FORM)**

The aim of this study is to determine the prevalence, antimicrobial resistance and risk factors of *Salmonella* in poultry production chain and humans in North-western Nigeria. Salmonella infection is responsible for 93.8 million cases of human gastroenteritis globally each year and poultry, is the common source and the main vehicles of *Salmonella* infection to humans.

The research work is part of the requirements for PhD thesis in molecular bacteriology and infection at the University of Copenhagen, Demark. The study will collect faecal sample from poultry, administer questionnaires to poultry farmers and collect faecal sludge from public toilets. All information obtained in the course of this study will be strictly confidential and you have the right to withdraw from the study at any point in time. If you are willing to participate, please complete the consent form below.

I ________________________________________________________ hereby give my consent to participate in the study having being fully explained regarding the study. I also note that I have the right to withdraw from the study at any point.

Signature of Participant and Date ______________________________

Signature of Researcher and Date _____________________________

Signature of Witness and Date_________________________________
